# Erratum to: Use of a MAIT-Activating Ligand, 5-OP-RU, as a Mucosal Adjuvant in a Murine Model of *Vibrio cholerae* O1 Vaccination

**DOI:** 10.20411/pai.v7i1.541

**Published:** 2022-09-13

**Authors:** Owen Jensen, Shubhanshi Trivedi, Jackson G. Cacioppo, Kelin Li, Jeffrey Aubé, J. Scott Hale, Edward T. Ryan, Daniel T. Leung

**Affiliations:** 1 Division of Infectious Diseases, Department of Internal Medicine, University of Utah School of Medicine, Salt Lake City, Utah; 2 Division of Microbiology & Immunology, Department of Pathology, University of Utah School of Medicine, Salt Lake City, Utah; 3 Division of Chemical Biology and Medicinal Chemistry, UNC Eshelman School of Pharmacy, University of North Carolina at Chapel Hill, Chapel Hill, North Carolina; 4 Division of Infectious Disease, Massachusetts General Hospital, Boston, Massachusetts; 5 Department of Medicine, Harvard Medical School, Boston, Massachusetts; 6 Department of Immunology and Infectious diseases, Harvard School of Public Health, Boston, Massachusetts

**ERRATUM TO: USE OF A MAIT ACTIVATING LIGAND, 5-OP-RU, AS A MUCOSAL ADJUVANT IN A MURINE MODEL OF VIBRIO CHOLERAE O1 VACCINATION**

**DOI:** 10.20411/pai.v7i1.525

**Reason:** The authors sincerely regret the inadvertent omission of Jackson G. Cacioppo as coauthor of this work. He has no additional conflicts of interest.

**Corrected version:** Owen Jensen^1,2^, Shubhanshi Trivedi^1^, Jackson G. Cacioppo^3^, Kelin Li^3^, Jeffrey Aubé^3^, J. Scott Hale^2^, Edward T. Ryan^4,5,6^, Daniel T. Leung^1,2*^

