# Supplementary material for: Use of a MAIT-Activating Ligand, 5-OP-RU, as a Mucosal Adjuvant in a Murine Model of Vibrio cholerae O1 Vaccination
Source: Pathog Immun. 2022 Aug 24;7(1):122–44. doi: 10.20411/pai.v7i1.525 (PMC9438945; doi:10.20411/pai.v7i1.525)
Supplement: Supplementary Figures and Table [file pai-7-122-s01.pdf]

PATHOGENS AND IMMUNITY
IN ASSOCIATION WITH CASE WESTERN RESERVE UNIVERSITY AND THE RICHARD J. FASENMYER FOUNDATION

SUPPLEMENTARY FIGURES AND TABLE

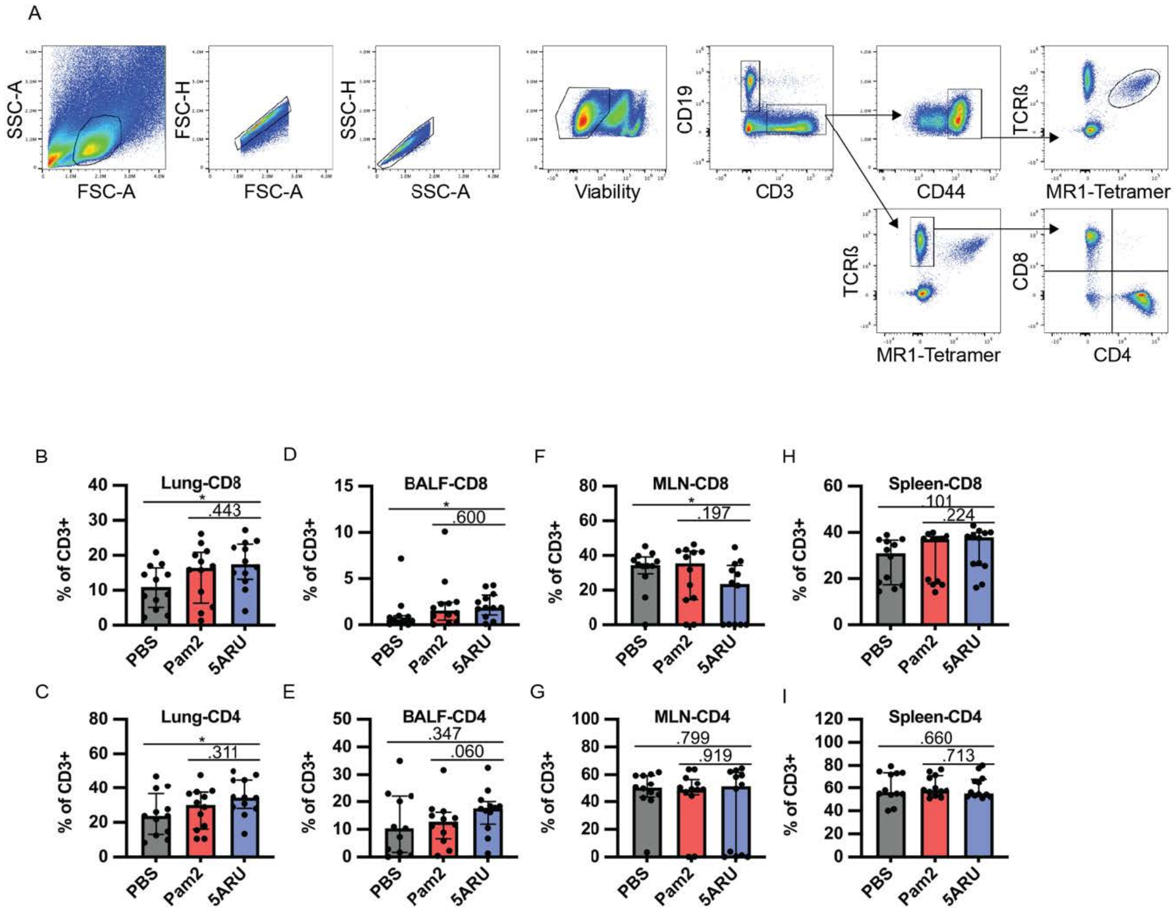

Supplementary Figure 1. No changes in non-MAIT T cell populations in live *V. cholerae* vaccination following 5-A-RU treatment. (A) Representative flow cytometry gating strategy for MAIT and non-MAIT T cells in mouse lungs vaccinated with live *V. cholerae*. (B-I) Frequency of CD8 and CD4 T cells as a percentage of total CD3+ cells in Lung (B-C), BALF (D-E), MLN (F-G), and spleen (H-I). Data are represented as Median with IQR from 3 independent experiments. n=11-12 mice per group. *$P < 0.05$, ** $P > 0.01$, *** $P > 0.001$, *** $P > 0.0001$ by 2-tailed Mann-Whitney U test.

Jensen O, Trivedi S, Li K, Aubé J, Hale JS, Ryan ET, Leung DT. Use of a MAIT-Activating Ligand, 5-OP-RU, as a Mucosal Adjuvant in a Murine Model of *Vibrio cholerae* O1 Vaccination. *Pathogens and Immunity*. 2022;7(1): 122-144. doi: 10.20411/pai.v7i1.525.

PATHOGENS AND IMMUNITY
IN ASSOCIATION WITH CASE WESTERN RESERVE UNIVERSITY AND THE RICHARD J. FASENMYER FOUNDATION

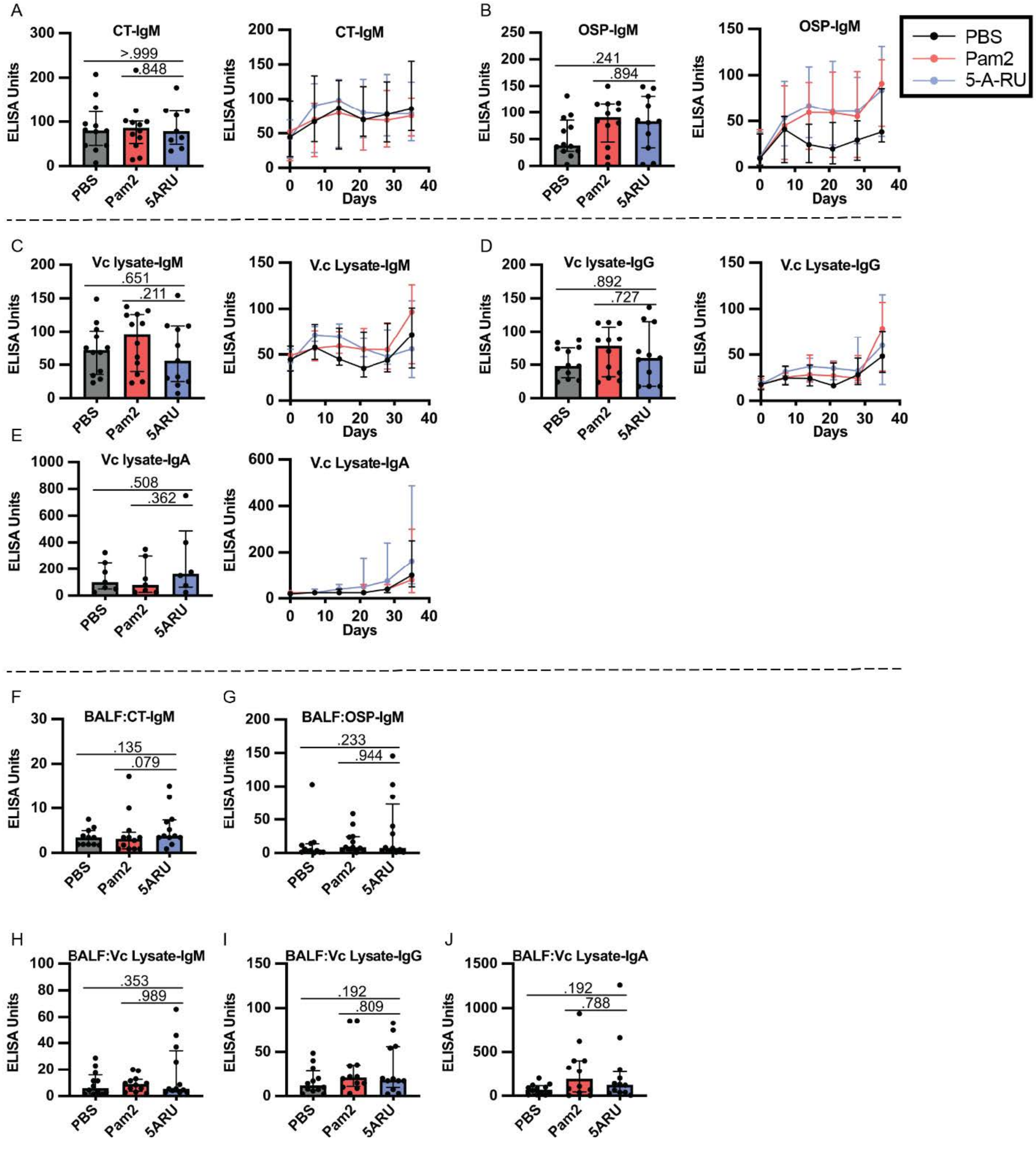

Supplementary Figure 2. Intranasal 5-A-RU has no effect on *V. cholerae*-specific antibody responses when administered with a live *V. cholerae* vaccination. Serum day 35 endpoint (left) and time-course (right) (A) CTIgM, (B) OSP-IgM, (C) *V. cholerae*-lysate-IgM, (D) *V. cholerae*-lysate-IgG, (E) *V. cholerae*-lysate-IgA ELISAs. (F-J) BAL fluid day 35 endpoint (F) CT-IgM, (G) OSP-IgM, (H) *V. cholerae*-lysate-IgM, (I) *V. cholerae*-lysate-IgG, and (J) *V. cholerae*-lysate-IgA ELISAs. Data are represented as ELISA units measured kinetically and normalized to positive control pooled serum from WT B6 mice intranasally vaccinated with live *V. cholerae*. Data are represented as Median with IQR from 3 independent experiments. n=11-12 mice per group. *P* values determined by 2-tailed Mann-Whitney *U* test.

Jensen O, Trivedi S, Li K, Aubé J, Hale JS, Ryan ET, Leung DT**.** Use of a MAIT-Activating Ligand, 5-OP-RU, as a Mucosal Adjuvant in a Murine Model of *Vibrio cholerae* O1 Vaccination. *Pathogens and Immunity*. 2022;7(1): 122-144. doi: 10.20411/pai.v7i1.525.

PATHOGENS AND IMMUNITY
IN ASSOCIATION WITH CASE WESTERN RESERVE UNIVERSITY AND THE RICHARD J. FASENMYER FOUNDATION

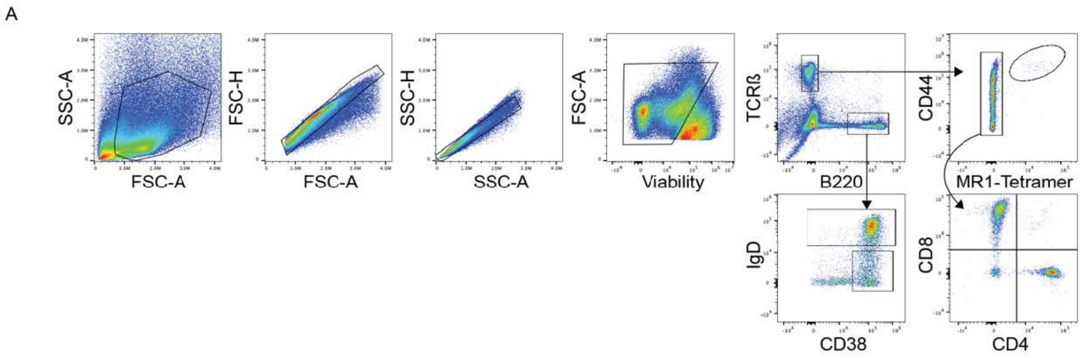

Supplementary Figure 3. Flow gating of T and B cell populations from intranasal V. cholerae OSP vaccination. (A) Representative flow cytometry gating strategy for MAIT cells, non-MAIT T cells and B cells in mouse lungs vaccinated with *V. cholerae* O1 Ogawa OSP:BSA.

Jensen O, Trivedi S, Li K, Aubé J, Hale JS, Ryan ET, Leung DT**.** Use of a MAIT-Activating Ligand, 5-OP-RU, as a Mucosal Adjuvant in a Murine Model of *Vibrio cholerae* O1 Vaccination. *Pathogens and Immunity*. 2022;7(1): 122-144. doi: 10.20411/pai.v7i1.525.

PATHOGENS AND IMMUNITY
IN ASSOCIATION WITH CASE WESTERN RESERVE UNIVERSITY AND THE RICHARD J. FASENMYER FOUNDATION

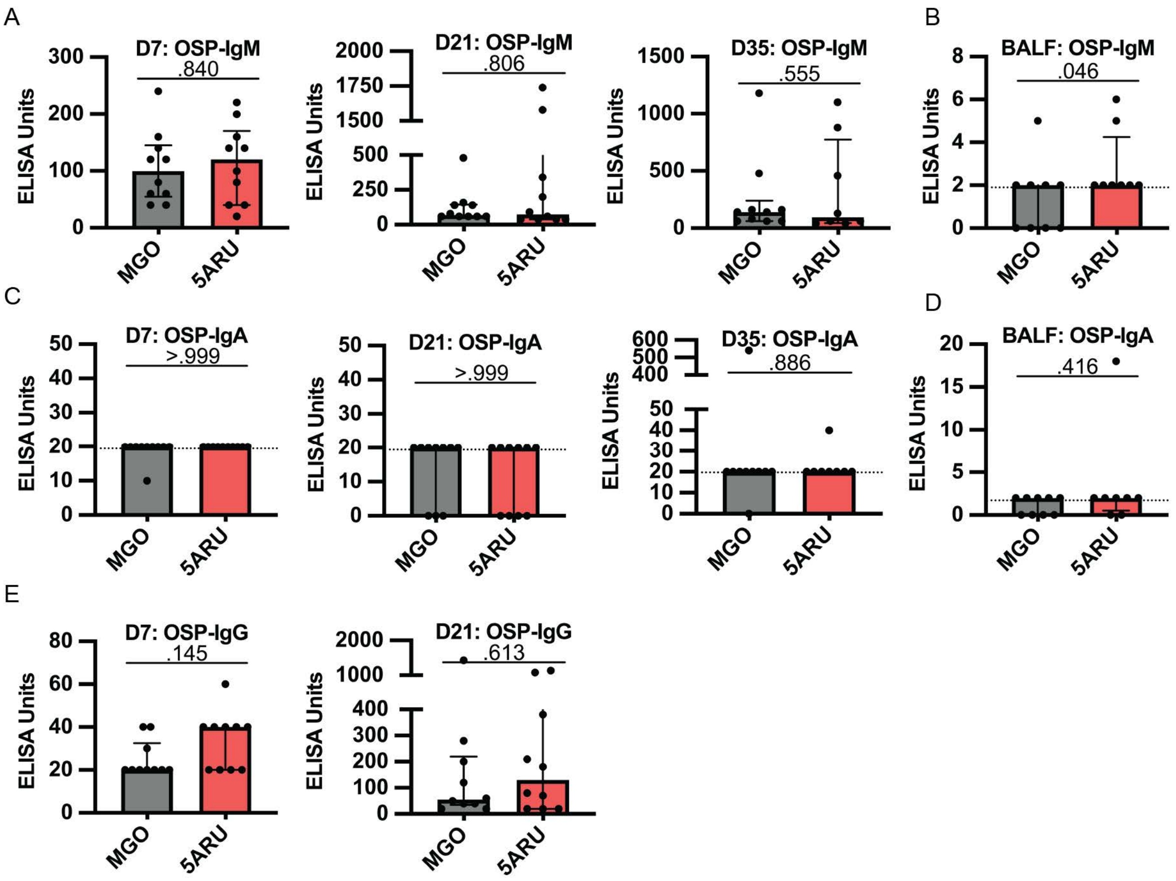

Supplementary Figure 4. Intranasal 5-A-RU has no effect on mucosal or systemic polysaccharide-specific IgM and IgA antibody responses. (A) Serum day 7 (left), day 21 (middle) and day 35 (right), and (B) BALF day 35 endpoint OSP-IgM ELISAs. (C) Serum day 7 (left), day 21 (middle) and day 35 (right), and (D) BALF day 35 endpoint OSP-IgA ELISAs. (E) Serum day 7 (left) and day 21 (right) OSP-IgG ELISAs. Data are represented as ELISA units measured kinetically and normalized to positive control pooled serum from WT B6 mice intranasally vaccinated with live *V. cholerae*. P values determined by 2-tailed Mann-Whitney *U* test. Dotted lines indicate Limit of Detection (LOD).

Jensen O, Trivedi S, Li K, Aubé J, Hale JS, Ryan ET, Leung DT**.** Use of a MAIT-Activating Ligand, 5-OP-RU, as a Mucosal Adjuvant in a Murine Model of *Vibrio cholerae* O1 Vaccination. *Pathogens and Immunity*. 2022;7(1): 122-144. doi: 10.20411/pai.v7i1.525.

PATHOGENS AND IMMUNITY
In Association with Case Western Reserve University and The Richard J. Fasenmyer Foundation

ELISA (Sample Tissue:Target)	MAIT Frequency (Tissue)	R squared	*P* Value
BALF:OSP-IgG	BALF	0.536	0.0389
BALF:OSP-IgG	Lung	0.1564	0.4377
Serum:OSP-IgG	BALF	0.2851	0.1728
Serum:OSP-IgG	Lung	0.01737	0.8327
Serum:OSP-IgM	BALF	0.03489	0.2169
Serum:OSP-IgM	Lung	0.7369	0.0287

Supplementary Table 1. OSP ELISA vs MAIT frequency. Simple linear regression analysis associating BALF and serum OSP:BSA IgG and IgA ELISA data and MAIT frequency from BALF and lungs in mice treated with *V. cholerae* OSP:BSA and 5-A-RU (Figures 4 and 5). BALF OSP-IgM ELISA data was omitted from analysis as few samples were above the LOD.

Jensen O, Trivedi S, Li K, Aubé J, Hale JS, Ryan ET, Leung DT**.** Use of a MAIT-Activating Ligand, 5-OP-RU, as a Mucosal Adjuvant in a Murine Model of *Vibrio cholerae* O1 Vaccination. *Pathogens and Immunity*. 2022;7(1): 122-144. doi: 10.20411/pai.v7i1.525.
